# Travel Route Planning with Optimal Coverage in Difficult Wireless Sensor Network Environment

**DOI:** 10.3390/s19081838

**Published:** 2019-04-17

**Authors:** Yu Gao, Jin Wang, Wenbing Wu, Arun Kumar Sangaiah, Se-Jung Lim

**Affiliations:** 1 College of Information Engineering, Yangzhou University, Yangzhou 225000, China; gaoyuyz@163.com; 2 Hunan Provincial Key Laboratory of Intelligent Processing of Big Data on Transportation, School of Computer & Communication Engineering, Changsha University of Science & Technology, Changsha 410000, China; wu_wenbing@163.com; 3 School of Information Science and Engineering, Fujian University of Technology, Fuzhou 350000, China; 4 School of Computing Science and Engineering, Vellore Institute of Technology (VIT), Vellore 632014, India; arunkumarsangaiah@gmail.com; 5 Liberal Arts & Convergence Studies, Honam University, Gwangju 622623624, Korea; limsejung@korea.ac.kr

**Keywords:** wireless sensor networks, mobile devices, travel route planning, particle swarm optimization, ant colony optimization

## Abstract

In recent years, wireless sensor networks (WSNs) have been widely applied to sense the physical environment, especially some difficult environment due to their ad-hoc nature with self-organization and local collaboration characteristics. Meanwhile, the rapid development of intelligent vehicles makes it possible to adopt mobile devices to collect information in WSNs. Although network performance can be greatly improved by those mobile devices, it is difficult to plan a reasonable travel route for efficient data gathering. In this paper, we present a travel route planning schema with a mobile collector (TRP-MC) to find a short route that covers as many sensors as possible. In order to conserve energy, sensors prefer to utilize single hop communication for data uploading within their communication range. Sojourn points (SPs) are firstly defined for a mobile collector to gather information, and then their number is determined according to the maximal coverage rate. Next, the particle swarm optimization (PSO) algorithm is used to search the optimal positions for those SPs with maximal coverage rate and minimal overlapped coverage rate. Finally, we schedule the shortest loop for those SPs by using ant colony optimization (ACO) algorithm. Plenty of simulations are performed and the results show that our presented schema owns a better performance compared to Low Energy Adaptive Clustering Hierarchy (LEACH), Multi-hop Weighted Revenue (MWR) algorithm and Single-hop Data-gathering Procedure (SHDGP).

## 1. Introduction

Wireless sensor networks (WSNs) have been widely applied recently to sense the physical environment, especially difficult environments due to their ad-hoc nature with self-organization and local collaboration characteristics. WSN, as a bond to connect the real world and digital systems, have attracted much attention over the past few decades [1,2,3,4]. In WSNs, sensors are generally deployed in the target areas by vehicles, such as planes, in a random manner. However, those target areas are usually in harsh environment, and once the sensors break down or exhaust their energy, they will be invalid because repair or battery replacement is impossible. After sensor deployment, they exchange the information with their neighbors and the WSN is formed rapidly. Due to the favorable characteristics of easy deployment and self-organization, as mentioned above, WSN can be seen everywhere in our daily life, such as environment monitoring [5], target surveillance [6,7], smart health [8,9] and many other intelligent systems [10,11,12,13].

Although WSNs are popular for some excellent characteristics, some challenges still trouble them [14,15]. The most severe one is the limited energy problem. Sensors are commonly powered by their carry-on batteries and battery replacement by manual operation is unrealistic, especially in some harsh environments [16]. Therefore, many researchers focus on the energy problems in WSNs. One of the efficient methods is routing protocol designing, and various energy efficient routing schemas are presented to prolong the lifetime of the network [17,18]. Traditional routing schema commonly focuses on the network with a fixed sink. In recent years, the development of an intelligent car has made it possible to gather mobile data. Intelligent cars are usually modified as mobile collectors by equipping multiple antennas so that they can collect the data from multiple objects simultaneously. 

**Figure 1:**
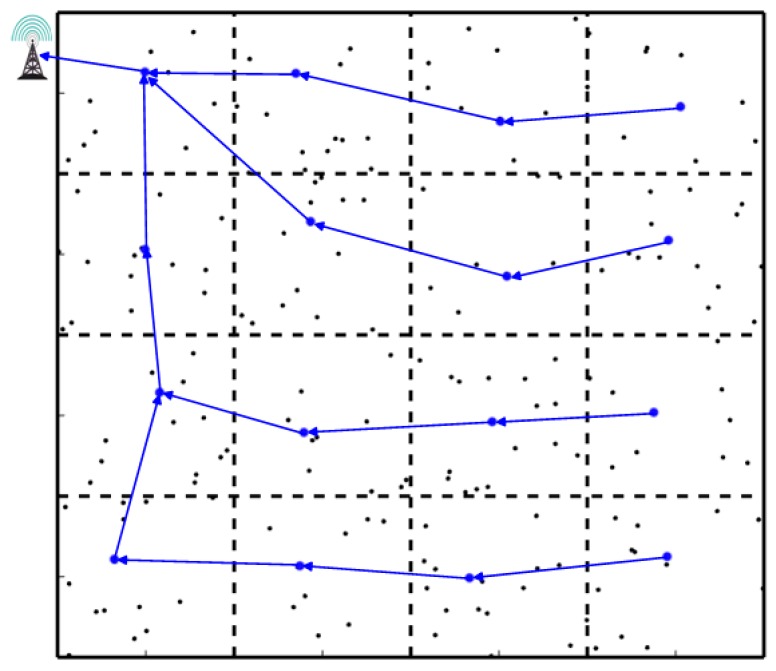
Clustering-based schema with a fixed sink.

Routing schemas for WSNs can be mainly classified into three categories as follows. Figure 1 describes the classic clustering-based schema with a fixed sink [19,20,21,22,23,24]. In this schema, the network is usually partitioned into several regions according to some special rules, and each region denotes a cluster. Each cluster chooses a leader with the most energy as its cluster head (CH). The data of member nodes are firstly transmitted to their corresponding CHs, and then CHs adopt multi-hop communication to upload the data to the remote sink. This schema has been widely used in some hierarchical routing protocols, such as Low Energy Adaptive Clustering Hierarchy (LEACH), Two-tier Data Dissemination (TTDD) algorithm and Hybrid, Energy-efficient, Distributed Clustering (HEED). 

**Figure 2:**
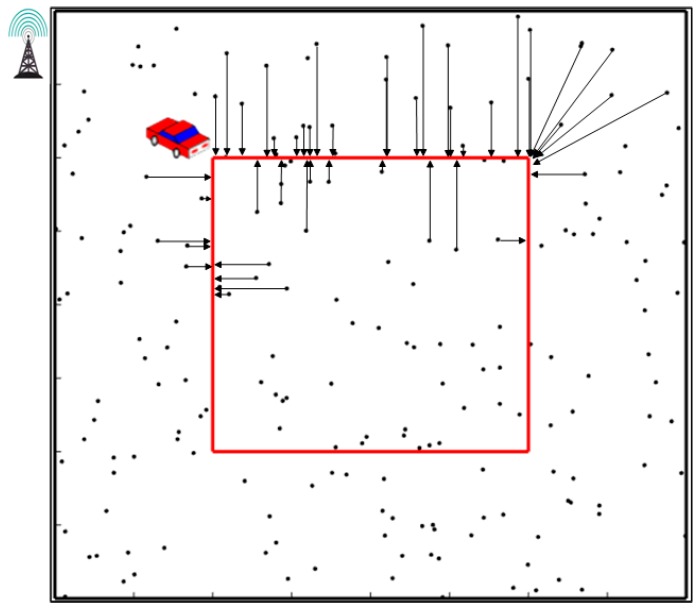
Data mule-based schema with a mobile collector.

Figure 2 describes the data mule-based schema with a mobile collector [25,26,27,28,29,30]. The mobile collector moves along a predefined path, and once a sensor enters its transmission range, it will stop for data gathering.

**Figure 3:**
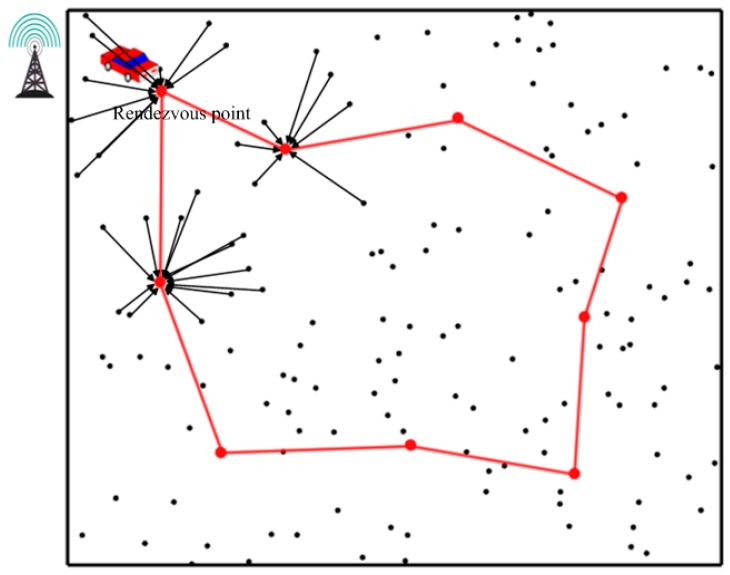
Rendezvous-based schema with a mobile data collector.

Figure 3 describes the rendezvous-based schema with a mobile data collector [31,32,33,34,35,36,37]. The rendezvous points are chosen in advance, and the mobile collector moves along a scheduled path to traverse all the rendezvous points. Data uploading only occurs when the mobile collector stops at any nearby rendezvous point. Data mule-based schema usually plans a long travel route for the mobile collector. Therefore, it results in serious network latency. Additionally, the changeless moving path is not flexible, and it is hard to balance the energy of different sensors. Rendezvous-based schema selects the sojourn points (SPs) for the mobile collector, and the total length of the travel route is related to the number and position of the selected SPs. Mobile collector in those schemas greatly enhances the performance of the network. However, the travel route of the mobile collector needs to be elaborately scheduled. 

In this paper, both the number and the position are considered to select a set of SPs to maximize the coverage rate of the sensors as well as minimize the overlapped coverage rate. We firstly illustrate the coverage problems in WSN, and then we figure out the suitable number of rendezvous points according to the transmission range. After that, the PSO algorithm is utilized to search the optimal position for the SPs. Finally, the shortest loop will be scheduled by the ACO algorithm for the mobile collector to traverse all the SPs. Most of sensors adopt single hop communication and only a few sensors especially some outliers inevitably utilize multiple hops communication.

The remaining sections of this paper are organized as follows. Section 2 illustrates some similar work related to our presented schema. The system model which contains the network model and energy model is demonstrated in Section 3. In Section 4, the coverage problem in WSN is analyzed and our presented TRP-MC is described in detail. Simulations are conducted and the results are analyzed in Section 5. Section 6 gives a discussion, and Section 7 concludes this paper.

## 2. Related Work

### 2.1. Clustering Based Schema with Fixed Sink

In this schema, a static sink is commonly deployed at one corner of the sensor field. Data are transmitted to the sink by multiple hops communication. One of the representative protocols of this schema is LEACH [19]. LEACH selects CHs in a random manner and sensors take turns to be the CHs for energy balancing. Sensors close to the same CH are classified into the same cluster. PEGASIS [20] adopts the chain structure to connect all the sensors for energy efficiency. The chain structure reduces the average communication distance between sensors, however, much data forwarding results in the heavy traffic burden of the network. HEED [21] firstly introduces the competition mechanism for clustering. Residual energy and the cost of intracluster communication are considered to calculate the competition range. This mechanism makes the CHs selection more reasonable. EEUC [22] also introduces the competition mechanism to divided sensors into clusters with different sizes. Clusters close to the sink have a smaller size so that the intracluster communication can be relieved and more energy can be used for data forwarding.

### 2.2. Data Mule Based Schema

In this schema, the network employs a mobile collector to walk through the sensor field along a regular way for data gathering. Once the mobile collector detects any sensor in its transmission, it will stop for data gathering using single hop communication. The mobile collector will return to the static sink at fixed periods for data uploading. Following are some representative protocols using this schema.

In reference [25], the authors presented a data dissemination method with a two-tier layer called TTDD. In TTDD, a grid structure is built by the source nodes. Firstly, the source node calculates the location of the virtual dissemination points and the sensors with the closet distance to the dissemination points are elected as dissemination nodes for data forwarding. Once sinks request information of the event, the query message is disseminated to the source node by forwarding of the dissemination nodes. Then, the data package is returned to the sink along with the previous dissemination path. If one disseminated node receives the same query message from a different sink, it will only forward one of them and drop the others.

In reference [26], the authors presented a data dissemination schema combined with multiple mobile sinks called MSDD. In MSDD, a two-level structure is constructed for data transmission. In the top level, mobile sinks cooperatively collect the information of source nodes utilizing a special schema. In the bottom level, the area of interest (AOI) is partitioned into several regions by grids. The dissemination nodes are introduced to assist sensors in forming different regions. A sink walks in the AOI randomly and once the event occurs, the query-driven routing path will be established. Simulation results validate the better performance of the presented method compared to TTDD.

In reference [27], the authors presented a new ideal to enhance the lifetime of the network by rotating the nodes with a heavy burden. Some mobile sensors are introduced to be the candidates for these “hot spots” and once the residual energy of these nodes is lower than a threshold value, the mobile sensors will exchange their location with the weak nodes.

In reference [28], the authors presented a dynamic routing schema with a mobile collector. They explore the routing method for WSN in a circular sensor field. The whole sensor field is partitioned into sectors for clustering, and a mobile data collector is utilized to move around the edge of the sensor field for data gathering. Additionally, this schema presented a dynamic routing method for intercluster data forwarding. The closest CH from the sink is chosen as its agent for direct communication, and then the other CHs are connected into a chain for data forwarding. 

### 2.3. Rendezvous Based Schema

In this schema, the moving trajectory, as well as the SPs of the mobile collector, are elaborately designed. Data transmission conducts only when the mobile collector stops at the SPs. Sensors far away from the SPs will transmit the data to a relay node close to the sojourn point in advance. Following are some representative protocols using this schema.

In reference [31], the authors presented a data collection schema that adopts sink mobility technology called MWR. In MWR, the compatible sensor pairs are elected to function as multiple antennas to simulate virtual multiple input and output (vMIMO). In order to address the problem of delay minimization for data gathering using multi-hop communication which has been validated to be an NP-hard problem, authors adopt integer linear program to formulate this problem. Some sensors are defined as polling points (PPs) for the mobile sink to access information collection. Authors used a heuristic approach to find the optimal positions of PPs, according to three crucial metrics, such as the compatibility of non-associated sensors, the capability of covering the non-associated neighbors, and the performance of moving length reducing.

In reference [32], a routing schema combined with clustering as well as dual data collection is presented, and it is called LBC-DUU. In LBC-DUU, the network is constructed with three-layer, they are sensor layer, CH layer, and mobile sink layer respectively. In sensor and CH layers, authors present a load balanced clustering (LBC) method to automatically partition the network into clusters by means of iteration. Then a sensor car is adopted as a mobile data collector to visit the polling points (PPs) which are handpicked. As the sensor car is equipped with two antennas, multiple CHs can upload their data to it together to reduce the time cost.

In reference [33], the authors presented a tree-based algorithm called MSMA. In MSMA, different structures of the topologies for different root nodes are scheduled in the initial phase and then the topology information is restored in the corresponding nodes. When the sink moves, the close nodes are elected as root nodes, and the topology is switched using the restored information. 

In reference [34], the authors planned the trajectory of the tour for multiple mobile sinks and presented SHDGP. In SHDGP, authors define polling points as the position where the data collectors can stop for data gathering. Each sensor can be chosen as a polling point or extra polling points can be explored by mobile sinks. Due to the unstable wireless communication, the neighbor set of each polling point will be confirmed by tentative communication. Then a spanning tree is generated by calculating the average cost of each polling point. Finally, the generated tree is broken up into subtrees according to the maximal tour length of each mobile sink.

Comparisons of different aspects of the mentioned protocols are listed in Table 1.

## 3. System Model

### 3.1. Fundamental Assumptions

In order to have a better illustration of our proposed TRP-MC and conduct the simulation conveniently, we present the following assumptions.

(1) All the sensors keep static after deployment, and once their energy is exhausted, they will be invalid.
(2) Sensors can adjust their communication distance within communication range and single hop communication are mainly utilized for data uploading.
(3) We define the sojourn points (SPs) as the places where the mobile collector stops for data gathering.
(4) A static sink is set at the corner of the sensor field, and during each round, the mobile collector will visit the sink once to upload its collected data.
(5) A mobile collector which is modified by an intelligent car is employed for data gathering. It travels through the sensor field and only stops at SPs which are elaborately selected for data gathering.

### 3.2. Network Model

**Figure 4:**
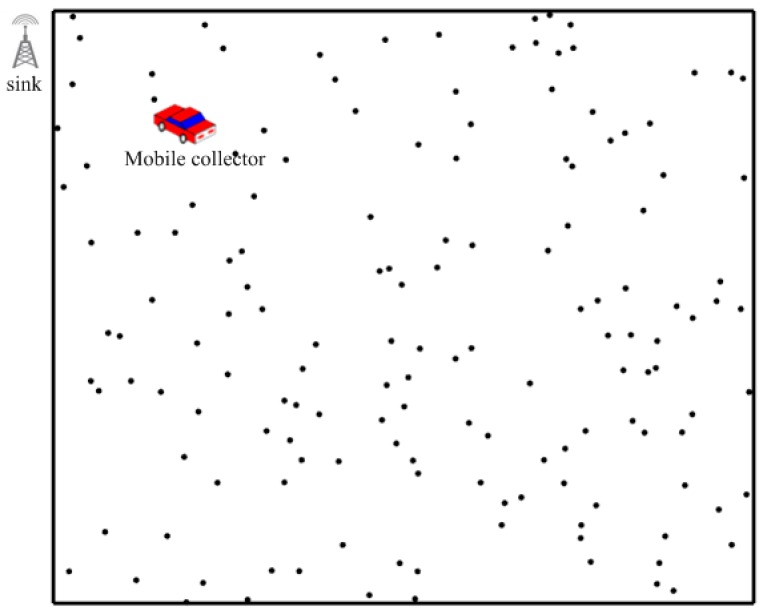
Network model.

The network model utilized in this paper is shown in Figure 4. We consider the sensor field as a rectangular area and plenty of sensors are deployed randomly in this area. There is no obstacle in the sensor field so that the mobile collector can move freely. Each round denotes the time for the mobile collector to traverse all the SPs. In each round, each sensor will upload a data package to the mobile collector respectively.

### 3.3. Energy Model

The energy consumption of WSNs is generally composed of many parts, such as monitoring, data storing, and data transmitting. However, the energy used for data transmission takes up a large proportion of the total energy consumption. Therefore, we only consider the energy consumption used in transmission in this paper. We use the same energy model as literature [38,39] adopted. The following formulas are used to calculate the energy consumption for sending *k*-bit data.

(1) $$E_{Tx}\left(k,dis\left(s_{m},s_{n}\right)\right)=E_{elec}\cdot k+\varepsilon_{amp}\cdot k$$

where $dis\left(s_{m},s_{n}\right)$ represents the distance between node *m* and node *n* and it can be calculated using Formula (2).

(2) $$dis\left(s_{m},s_{n}\right)=\sqrt{{\left(x_{m}-x_{n}\right)}^{2}+{\left(y_{m}-y_{n}\right)}^{2}}$$

$E_{elec}$ represents the energy to run the transmission circuit. $\varepsilon_{amp}$ denotes the energy consumption for the amplifier to strengthen the signal for further transmission and it can be further divided into Formula (3).

(3) $$\varepsilon_{amp}=\{\begin{array}{c} \varepsilon_{fs}\cdot dis^{2}\;if\;dis\leq d_{0}\\ \varepsilon_{mp}\cdot dis^{4}\;if\;dis>d_{0} \end{array}$$

where $\varepsilon_{fs}$ represents the power for close range communication and $\varepsilon_{mp}$ represents the power for long distance communication. Those two schemas are also called free space model and multi-path fading model, respectively. The threshold value $d_{0}$ is used to judge which model should be adopted and it can be calculated as:

(4) $$d_{0}=\sqrt{\frac{\varepsilon_{fs}}{\varepsilon_{mp}}}$$

The energy dissipates for receiving *k*-bit data can be figured out by Formula (5).

(5) $$E_{Rx}=E_{elec}\cdot k$$

## 4. Our Presented TRP-MC Algorithm

### 4.1. Coverage Problem Formulation

**Figure 5:**
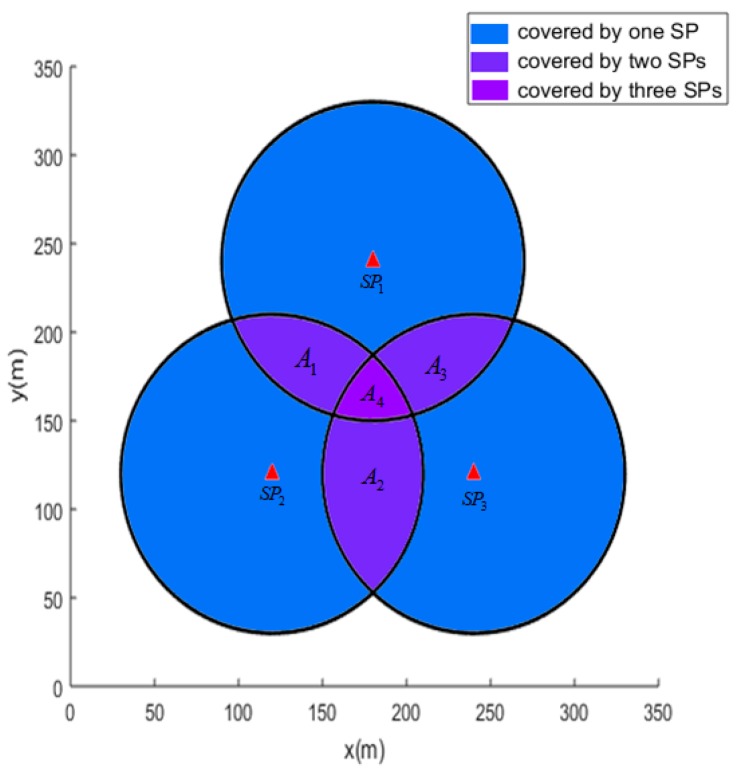
Covered areas of the mobile collector.

In this section, we first transform the travel route planning problem of the mobile collector into the coverage problem of SPs. As we all know, two sensors can communicate with each other only when they are both in their communication range. Although the mobile collector possesses a better performance than the common sensors, their valid communication ranges are the same. We assume that the mobile collector only stops for data gathering because moving data gathering will result in a high package loss rate and much energy will be dissipated for retransmission. The places where the mobile collector stops are called SPs. When the mobile collector stops at one SP, it can communicate with the sensors within its transmission range. We define the covered area as the place where the mobile collector can communicate with when it stops at SPs. A coverage sample is shown in Figure 5.

Where $SP_{1}$, $SP_{2}$ and $SP_{3}$ represent three different covered areas of SPs, respectively. As Figure 5 describes, the area $A_{1}$, $A_{2}$ and $A_{3}$ are covered by 2 SPs and $A_{4}$ is covered by 3 SPs simultaneously. We define those areas which are covered by at least one SP as covered area, and those areas which are covered by more than one SP as overlapped covered areas. One sensor can upload its monitored data to the mobile collector if it is deployed at any covered area of the SP. However, overlapped covered areas dissipate the resource of the mobile collector and increase the travelling distance of the mobile collector. The mobile collector will work more efficiently if the overlapped covered areas are decreased.

**Figure 6:**
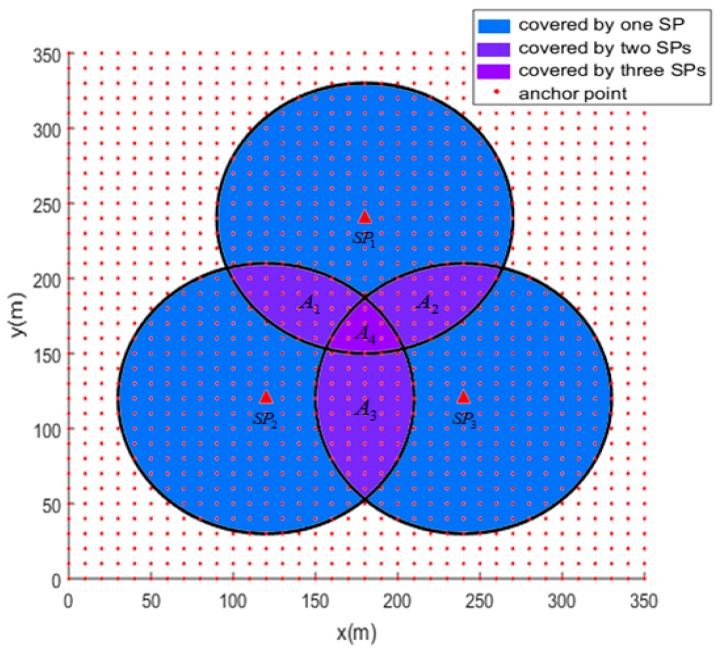
Anchors for coverage rate calculation.

Due to the round shape of each covered area, it is difficult to calculate the overlapped covered areas, especially the areas covered by multiple SPs. Hence, we introduce anchor points to assist in calculating the overlapped coverage rate. The anchor points are virtual points evenly distributed in the sensor field, as is shown in Figure 6.

Each anchor point can calculate its distance to the SP to confirm whether it is covered by the SP. Therefore, we define the coverage rate and overlapped coverage rate using Formulas (6) and (7).

(6) $$r_{cover}=\frac{n_{cover}}{n}$$

(7) $$r_{overlap\_cover}=\frac{n_{overlap\_cover}}{n_{cover}}$$

where n denotes the number of anchor points. $n_{cover}$ and $n_{overlap\_cover}$ denote the anchor points cover by at least one SP and more than one SPs, respectively. Additionally, the accuracy of $r_{cover}$ and $r_{overlap\_cover}$ is corresponding to the density of anchor points. In Figure 5, the coordinates of SPs $SP_{1}$, $SP_{2}$ and $SP_{3}$ are (180,240), (120,120), and (240,120), respectively. The corresponding transmission range is 90 and the density of the anchors is one. The coverage rate and the overlapped coverage rate are 0.5214 and 0.1718, respectively.

In our presented TRP-MC, we regard the sensor nodes as the anchor points mentioned above. The TRP-MC can be formalized after all the SPs are selected. When the mobile collector stops at the SPs, sensors which are in the transmission range of the mobile collector are called the neighbors of SPs. Our goal is to use a fixed number of SPs to cover as many sensors as possible and the overlapped coverage rate is as low as possible at the same time. The object of TRP-MC can be transformed into mixed-integer programming using Formula (8).

(8) $$Maximizer\;r_{cover}\;\&\&\;Minimize\;r_{overlap\_cover}$$

where $r_{cover}$ and $r_{overlap\_cover}$ can be calculated using Formulas (9) and (10).

(9) $$r_{cover}=\frac{\sum_{i=1}^{n}c_{i}}{n}$$

(10) $$r_{overlap\_cover}=\frac{\sum_{i=1}^{n}o_{i}}{\sum_{i=1}^{n}c_{i}}$$

where *n* is the number of sensors, $c_{i}$ and $o_{i}$ are illustrated in Formulas (11) and (12), respectively.

(11) $$c_{i}=\{\begin{array}{c} 1\;if\;sensor\;i\;is\;covered\;by\;at\;least\;one\;SP\\ 0\;otherwise \end{array}$$

(12) $$o_{i}=\{\begin{array}{c} 1\;if\;sensor\;i\;is\;covered\;by\;more\;than\;one\;SPs\\ 0\;otherwise \end{array}$$

### 4.2. Hexagon Division

**Figure 7:**
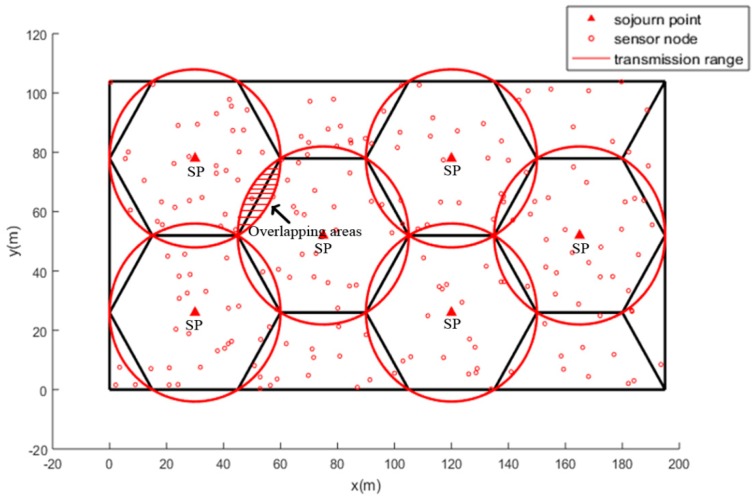
Hexagon division.

We consider the sensor field is covered by a fixed number of SPs and one of the efficient methods has been discussed in reference [40] for sensor deployment. The authors use a regular hexagon to divide the sensor field and the length of the regular hexagon is set to the transmission range. Therefore, in each regular hexagon, only one node needs to keep active and the other nodes can stay in sleep mode momentarily. This method makes full use of the perceived range of sensor so that the active nodes in the network are decreased. In the same way, we can introduce a regular hexagon to select the SPs for a mobile collector. Figure 7 shows the selection of SPs using a regular hexagon. The side length of the regular hexagon is the transmission range of sensors and each sensor in the covered area can upload its data to its corresponding SP. This approach decreases the number of SPs and the travel route length is reduced. However, the areas covered by two adjacent SPs still have few overlaps.

### 4.3. Coverage Optimization Using PSO

In order to further optimize the number of SPs and the travel route length, we introduce PSO to find the best position for the SPs. Particle swarm optimization (PSO) is a searching algorithm inspired by the hunting process of birds [41]. It can achieve the near-optimal solution in the searching space by iteration. As tour scheduling for the mobile collector has been verified as an NP-hard problem, it is difficult to figure out the optimal solution. Therefore, PSO is very suitable for the tour scheduling in WSNs. We use the virtual particles to represent the location of SPs, and each particle denotes a whole solution for the SPs selection. Due to the fixed dimension of particles in PSO, first we need to confirm the number of SPs. First, we assume that SPs can make full use of the wireless communication space and the number of SPs can be calculated using Formula (13). Different number of SPs will be discussed in the next section.

(13) $$spn=\lceil \frac{S}{\pi r^{2}}\rceil$$

where *S* denotes the total area of the sensor field, and *r* denotes the transmission range of the sensors. Then we can construct the particles using a matrix with pn×(2·spn) dimension which is shown as Formula (14) and pn denotes the number of particles.

(14) $$P=\left[\begin{array}{l} p^{1}\\ p^{2}\\ p^{2}\\ \vdots \\ p^{pn} \end{array}\right]=\left[\begin{array}{c} x_{sp_{1}}^{1},y_{sp_{1}}^{1},x_{sp_{2}}^{1},y_{sp_{2}}^{1}\ldots \ldots x_{spn}^{1},y_{spn}^{1}\\ x_{sp_{1}}^{2},y_{sp_{1}}^{2},x_{sp_{2}}^{2},y_{sp_{2}}^{2}\ldots \ldots x_{spn}^{2},y_{spn}^{2}\\ x_{sp_{1}}^{3},y_{sp_{1}}^{3},x_{sp_{2}}^{3},y_{sp_{2}}^{3}\ldots \ldots x_{spn}^{3},y_{spn}^{3}\\ \vdots \\ x_{sp_{1}}^{pn},y_{sp_{1}}^{pn},x_{sp_{2}}^{pn},y_{sp_{2}}^{pn}\ldots \ldots x_{spn}^{pn},y_{spn}^{pn} \end{array}\right]$$

where a 2-tuple $\left(x_{sp_{i}}^{k},y_{sp_{i}}^{k}\right)$ denotes the location of the *i*-th SP in the *k*-th particle. We set the restrictions for the moving speed of the particles in each dimension and the location of the particles. The restrictions are shown as Formulas (15) and (16).

(15) $$\mathrm{restriction}\left(v_{i}^{k}\right)=\left[-20,20\right]$$

(16) $$\mathrm{restriction}\left(x_{sp_{i}}^{k},y_{sp_{i}}^{k}\right)=\left[0,L\right]$$

where $v_{i}^{k}$ denotes the speed of the *i*-th dimension of the *k*-th particles. Formula (15) ensures that the particles will not move too fast and Formula (16) guarantees the particles will not move away from the sensor field. Next, we define the fitness function for the PSO. As $r_{cover}$ and $r_{overlap\_cover}$ are both positive numbers, we adopt Formula (17) which is transformed from Formula (8) as the fitness function and our goal is to minimize the fitness function.

(17) $$\mathrm{Fitness}=\frac{r_{overlap\_cover}}{r_{covered}}$$

Then we execute the PSO algorithm using the following steps.

**Step 1:** We initialize the particles *P* and the speed of the particles *v* with random numbers and they both satisfy the above restrictions.

**Step 2:** The value of the fitness function is calculated according to Formula (17). Then each particle compares the current fitness value with its previous optimal fitness value and chooses the smaller one as its local optimal solution which is denoted as $L_{best}$. In the same way, the previous optimal fitness value of all particles is compared with its current fitness value and the smaller one is chosen as the global optimal solution which is denoted as $G_{best}$.

**Step 3:** Then, we use Formulas (18) and (19) to update the speed and the location of the particles respectively.

(18) $$V\left(t+1\right)=\lambda V\left(t\right)+\alpha \cdot \mathrm{random}\cdot \left(L_{best}-P\left(t\right)\right)+\beta \cdot \mathrm{random}()\cdot \left(G_{best}-P\left(t\right)\right)$$

(19) P(t+1)=P(t)+V(t+1)

where λ represents the inertia coefficient and the function of random() is to generate a random number which is between zero and one. α and β are two weight coefficients, and they satisfy α+β=1.

**Step 4:** After updating the parameters of the particles, the bounds checking is conducted. If the speed exceeds the restriction as Formula (15) describes, we set it as the boundary value. If the location of the particle exceeds the boundary of the sensor field as Formula (16) describes, we set it as the boundary value. 

**Step 5:** Next, the algorithm returns to step 2 and iterates to the maximal number of iterations.

**Figure 8:**
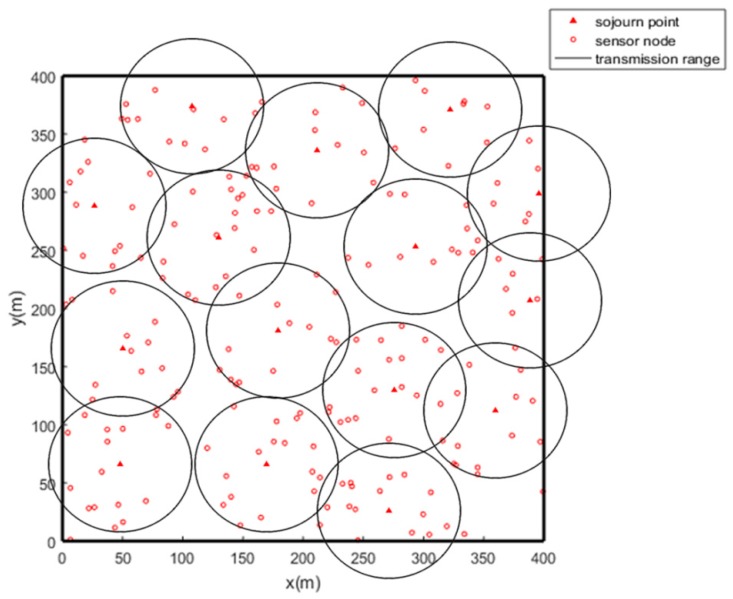
SPs selection using PSO.

Ultimately, we achieve the optimal solution for the SPs selection as $G_{best}$ shows. One sample after executing PSO is shown in Figure 8. As Figure 8 shows, most of the sensors are covered by only one SP and only a few sensors are covered by multiple SPs. Inevitably, very few sensors may not be covered by any SPs especially when they are outliers. 

For those uncovered sensors, we could only introduce multi-hop transmission for data delivering. Once a sensor finds that it is not covered by any SP, it will choose the closest relay node which is covered by the nearby SP. Although multi-hop communication still exists in the network, it has a slight effect on the performance of the network because only a few sensors will use it.

### 4.4. Travel Path Planning Using ACO

After all the SPs are selected, the shortest loop is scheduled by ACO to connect all the SPs. ACO is a metaheuristic algorithm inspired by the food-seeking of ants [42]. Ants secrete pheromones on their moving path and the shorter path will be traveled by more ants. Then, higher concentration of pheromones will be focused on those paths. Therefore, ants can seek a shorter path by referring to the experience of other ants. We regard the network as a complete undirected graph which is denoted by G=<V,E>, where V denotes the set of SPs and E denotes the virtual links between every two SPs. We use the matrix A with dimension m×spn to record the journey of ants, where m denotes the number of ants and spn denotes the number of SPs. Matrix T with dimension spn×spn is used to record the pheromone concentration of the virtual link. ACO algorithm is executed using the following steps.

**Step 1:** We randomly distribute m ants in SPs and initialize matrix A.

**Step 2:** Then, we calculate the possibility of selecting SPs which are not visited as the next destination for each ant, according to the pheromone concentration and their distance. Ants visit all those SPs according to the possibility and record the journey in matrix A. The possibility of SPs which are not visited can be calculated by Formula (20).

(20) $$p_{ij}^{k}\left(t\right)=\{\begin{array}{c} \frac{\varphi_{ij}^{\alpha }\left(t\right)\cdot \mu_{ij}^{\beta }\left(t\right)}{\sum_{m\in next}\varphi_{im}^{\alpha }\left(t\right)\cdot \mu_{im}^{\beta }\left(t\right)}\;if\;j\in next\\ 0\;otherwise \end{array}$$

where $p_{ij}^{k}\left(t\right)$ represents the possibility of selecting the path from $SP_{i}$ to $SP_{j}$ for the *k*-th ant in the *t*-th iteration. $\varphi_{ij}$ denotes the pheromone concentration of the path from $SP_{i}$ to $SP_{j}$ and $\mu_{ij}$ denotes the reciprocal of the distance from $SP_{i}$ to $SP_{j}$. α and β denote two different control factors for pheromone concentration and inspired factor. The set *next* denotes the SPs that the ant has not visited.

**Step 3:** Next, we calculate the journey of each ant using Formula (21). Since our goal is to minimize the tour length of the mobile collector, we only evaluate the journey on its length.

(21) $$L\left(a^{k}\right)=\underset{i,j\in SPs,i\neq j}{\sum }l_{ij}\cdot b_{ij}$$

where $l_{ij}$ denotes the distance from $SP_{i}$ to $SP_{j}$ and $b_{ij}$ can be illustrated using Formula (22).

(22) $$b_{ij}=\{\begin{array}{c} 1\;if\;path\;from\;SP_{i}\;to\;SP_{j}\;is\;contained\;on\;k-th\;ant^{\prime }s\;journey\\ 0\;otherwise \end{array}$$

**Step 4:** Finally, we update the concentration of the pheromone using the following formulas.

(23) $$\varphi_{ij}\left(t+1\right)=\left(1-\eta \right)\cdot \varphi_{ij}\left(t\right)+\Delta \varphi_{ij}$$

(24) $$\Delta \varphi_{ij}=\overset{m}{\underset{k=1}{\sum }}\Delta \varphi_{ij}^{k}$$

(25) $$\Delta \varphi_{ij}^{k}=\{\begin{array}{c} \frac{Q}{L\left(a^{k}\right)}\;if\;the\;k-th\;ant\;pass\;path_{ij}\\ 0\;otherwise \end{array}$$

where η denotes the volatilization rate of pheromone and *Q* denotes the total amount of pheromone secreted by one ant during one whole travel.

**Step 5:** Steps 1–4 are repeatedly executed until the algorithm achieves its maximal iteration number.

**Figure 9:**
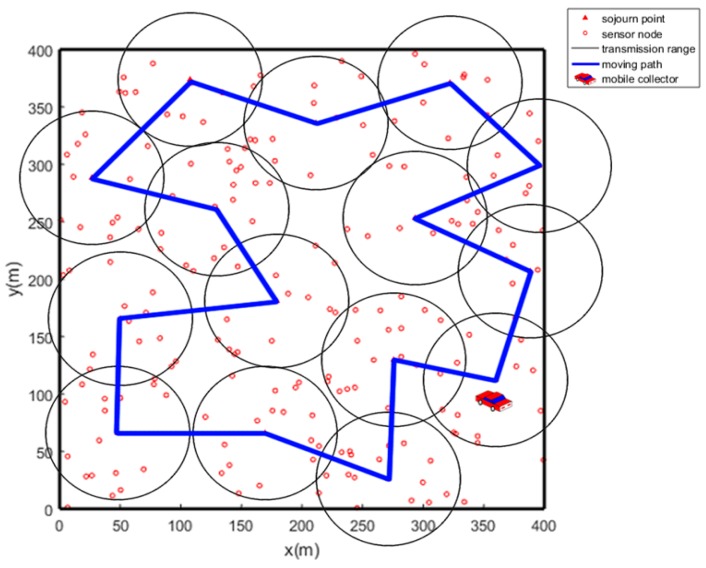
Path planning using ACO.

The same sample after executing ACO is shown in Figure 9.

## 5. Performance Evaluation

### 5.1. Parameters for PSO and ACO

We firstly execute the PSO algorithm for SPs selection, and different values of the PSO parameters are tried to explore the optimal performance of the algorithm. Then the ACO algorithm is carried out many times with different ACO parameters to find the shortest loop. All the optimal parameters for PSO and ACO are listed in Table 2.

### 5.2. Network Parameters and Settings

We adopt Matlab as the simulator to test the performance of our presented TRP-MC. We also compare it with some similar work, such as LEACH, SHDGP, and MWR and analyze the simulation results. Some relevant parameters of the network are described in Table 3.

We use the same network model to execute LEACH, SHDGP, and MWR to equally evaluate their performance. Following are some settings for the compared methods.

**LEACH:** The number of cluster heads in LEACH is the same as nsp and a static sink is placed in the center of the network. We assume that the transmission range of sensors in LEACH is big enough that every two sensors in the network can communicate with each other.

**SHDGP:** We only adopt the single mobile collector pattern in SHDGP. The speed of the mobile collector and the sojourn time of data uploading are the same as TRP-MC. Transmission range of sensors is also the same as that in TRP-MC.

**MWR:** The number of polling points in MWR is the same as the number of SPs in TRP-MC. The transmission range of MWR and TRP-MC are the same. In the same way, the mobile data collector owns the same properties as the mobile collector in TRP-MC.

### 5.3. Comparison of Energy Consumption

**Figure 10:**
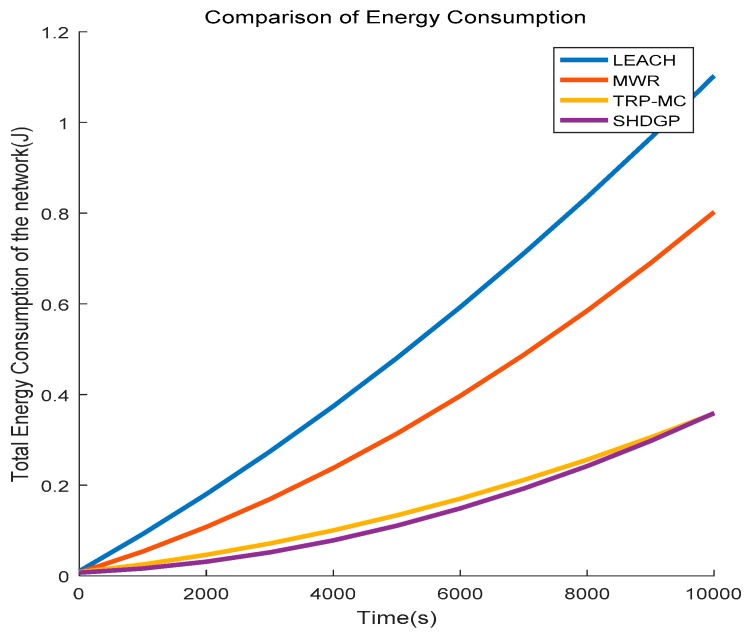
Comparison of energy consumption between different algorithms.

The energy consumption of the network using different methods is firstly compared. As Figure 10 demonstrates, the energy consumption of the four algorithms increases while SHDGP obtains a better performance compared to the other three algorithms. SHDGP achieves a little improvement in the performance of energy consumption compared to TRP-MC. However, energy consumption in SHDGP increases more rapidly than TRP-MC. When the time is about 10000 s, SHDGP consumes the same energy as TRP-MC, and we can infer that TRP-MC will achieve better performance on energy consumption than SHDGP after 10,000 s. LEACH consumes the most energy because it is a multi-hop based routing schema and many cluster heads have to transmit data packages to the fixed sink via long-distance communication. MWR, TRP-MC, and SHDGP all adopt mobile collector to gather the data, therefore, their energy consumption decreases in various degrees compared to LEACH. MWR defines compatible pairs for data forwarding while the communication between data collector and compatible pairs are much smaller than LEACH. TRP-MC and SHDGP are both single-hop based schemas, therefore, they both economize much energy and have similar performance in aspects of energy consumption.

### 5.4. Comparison of Network Lifetime

**Figure 11:**
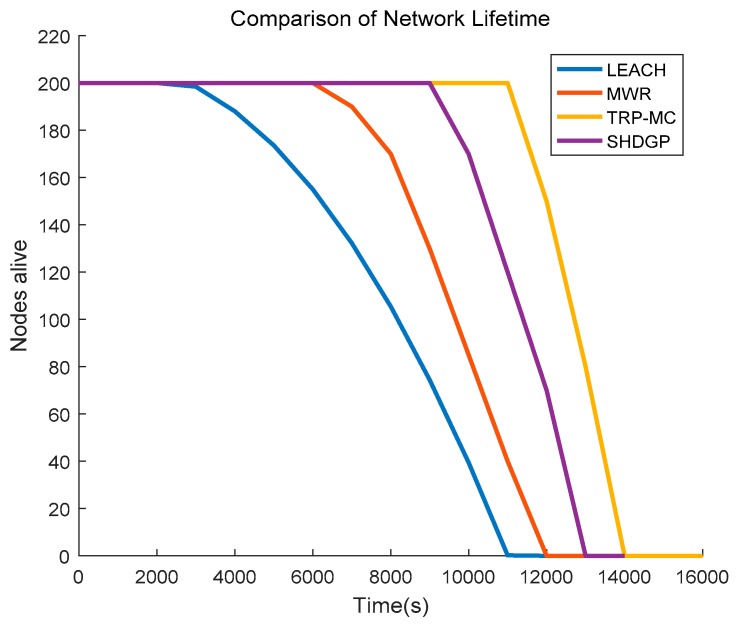
Comparison of network lifetime between different algorithms.

We next explore the lifetime of the network after executing different algorithms. Generally, the network lifetime is defined as the time when nodes in the network begin to die. The simulation result is illustrated in Figure 11. In LEACH, the first node dies at about 2300 s and the other three algorithms are much superior to LEACH in aspects of network lifetime. Uneven energy dissipation of nodes, especially the cluster heads and super-long-distance communication, result in the premature death of sensors in LEACH. The adoption of mobile collector greatly decreases the communication distance between the source node and the gatherer. Therefore, the network lifetime of those schemas with a mobile data collector is prolonged in various degree compared to LEACH. Multi-hop communication in MWR increases the burden of compatible pairs which function as the forwarders and that is the primary reason for the short lifetime of MWR. Although TRP-MC and SHDGP both adopt single-hop communication, TRP-MC minimizes the overlapped nodes of SPs and further improves the gather efficiency of the mobile collector.

### 5.5. Comparison of Travel Route Length

**Figure 12:**
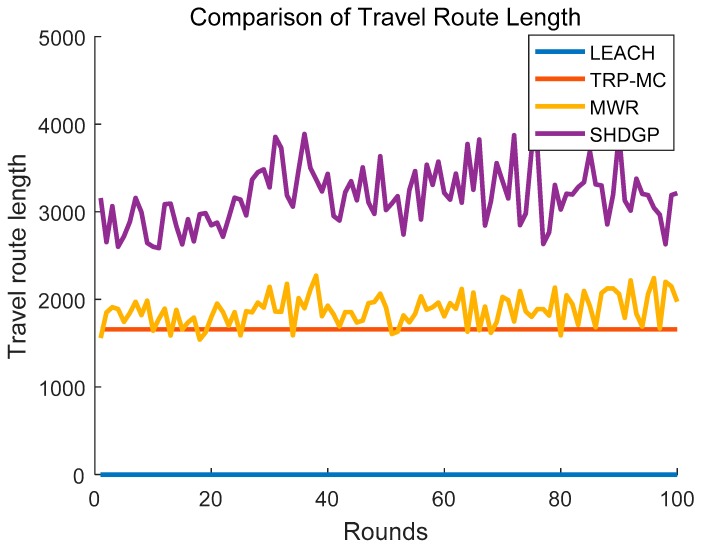
Comparison of travel route length between different algorithms.

Generally, the mobile collector is deployed in a converted intelligent car and its velocity is limited by its physical features. Therefore, the scheduled route length has a significant influence on the performance of the network especially network latency. We define the round as the period which denotes the time for the mobile collector to visit all the SPs for data gathering. Different algorithms are executed in many rounds respectively to compare the scheduled route length. The simulation result is described in Figure 12. LEACH generally adopts a static sink to gather information, and we set its travel route length is zero. The travel route length in TRP-MC is constant because the coverage rate will not change once the network model and transmission range are determined. Therefore, TRP-MC only needs to be executed once and the energy consumption used for control messages is greatly decreased. SHDGP plans the longest travel route path in each round because it constructs a tree structure to traverse all the polling points instead of a loop and many paths have to be visited repeatedly.

### 5.6. Study on the Nnumber of SPs

**Figure 13:**
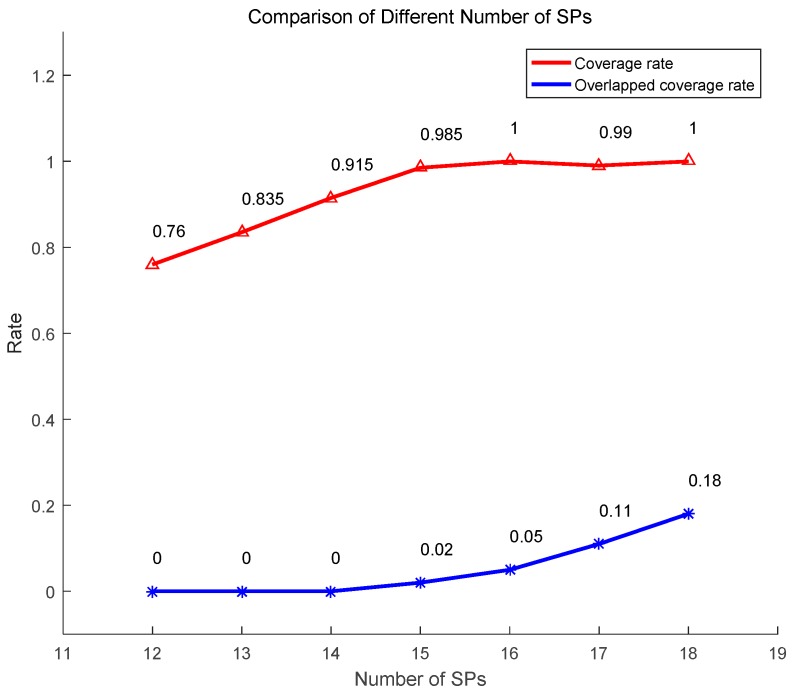
Comparison of different numbers of SPs.

As we mentioned above, we first calculate the number of SPs according to the transmission range and network size. However, other numbers of SPs are also feasible and they will result in different impacts. We use the same network to execute TRP-MC with different numbers of SPs. We first focus on the coverage rate and overlapped coverage rate of sensors. The simulation result is demonstrated in Figure 13. We can see that with the number of SPs increasing, the coverage rate also raises. When the number of SPs exceeds 15, the coverage rate almost does not change. The overlapped coverage rate hardly changes when the number of SPs is under 15. Therefore, 15 is a suitable number of SPs for the applied network model. 

## 6. Discussion and Future Work

Travel route planning is a crucial procedure among the routing protocol designing with a mobile collector. The scheduled route has a significant influence on the performance of the network. If the scheduled path is too long, it will take the mobile collector much time for traveling and the network will experience serious network latency. If the scheduled path is too short, many sensors may not communicate with the mobile collector directly so that much energy will be wasted for data forwarding. Therefore, it is difficult to schedule a short path as the coverage rate of sensors is high at the same time. In this paper, we not only focus on the coverage rate of the SPs but also take the overlapped coverage rate into consideration. The overlapped covered sensors waste the efficiency of the usage of the mobile collector. Additionally, the overlapped coverage rate is generally in inverse proportion to the coverage rate which accelerates the convergence of the fitness function of our proposed schema.

A single mobile collector contributes limited improvement in the performance of the network. With the incessant improvement of the software and hardware, the cost of the mobile collector is decreasing and more and more researchers transform their attention to WSNs with multiple mobile devices. Following are some problems which need to be addressed for multiple mobile sink-based routing protocol. Firstly, a communication mechanism should be designed for communication between the mobile collectors. Then, a cooperative mechanism which contains the moving paths and the target areas division for each collector should be elaborately designed. Additionally, the speed of the mobile collectors should also be controlled to confirm that the data of the network is synchronized. Our future work will mainly focus on the problems mentioned above and further improve the performance of the network. 

## 7. Conclusions

Travel route planning plays a significant role in protocol designing adopts the mobile collector. In this paper, we transform the path planning problem into the coverage problem in WSN and optimize the SPs for the mobile collector. We first formulate the problem as mixed-integer programming, and then the PSO algorithm is utilized to optimize the position of the SPs to cover more sensors. Additionally, we also take the overlapped coverage rate into consideration to accelerate the convergence of the algorithm. After the SPs are determined, the ACO algorithm is introduced to connect all the SPs into a loop with the shortest length. Plenty of simulations prove the effectiveness of our presented schema in aspects of energy consumption, network lifetime and network latency.

## Figures and Tables

**Table 1:** Comparison of some mentioned routing protocols.

Protocol Name	Year	Targets	Routing Schema	Sink Type	Clustering	Topology Control	Contributions
LEACH [19]	2000	Energy efficient	Clustering-based	Single static sink	True	Distributed	Hierarchical routing
PEGASIS [20]	2003	Energy efficient	Clustering-based	Single static sink	False	Distributed	Chain structure routing
HEED [21]	2004	Energy efficient, energy balancing	Clustering-based	Single static sink	True	Distributed	Competitional CHs selection
EEUC [22]	2005	Energy balancing	Clustering-based	Single static sink	True	Distributed	Competitional CHs selection
TTDD [25]	2005	Efficient data delivery	Data mule based	Multiple mobile sinks	False	Query driven	Virtual grid division, dissemination nodes selection
MSDD [26]	2014	Energy efficient	Data mule based	Multiple mobile sinks	False	Query driven	Virtual grid division, dissemination nodes selection
MNTL-MNR [27]	2012	Energy balancing	Data mule based	Single static sink	False	Distributed	Adoption of mobile CHs
Wang et al. [28]	2017	Energy efficient, energy balancing	Data mule based	Single mobile sink	true	Centralized	Special clustering, dynamic routing
MWR [31]	2016	Minimize network latency	Rendezvous-based	Single mobile sink	False	Centralized	Combining clustering whit vMIMO
LBC-DUU [32]	2015	Energy efficient, energy balancing	Rendezvous-based	Single mobile sink	True	Distributed	Three-layer routing structure
MSMA [33]	2015	Energy efficient	Rendezvous-based	Single mobile sink	False	Distributed	Tree-structure routing
SHDGP [34]	2013	Tour length scheduling	Rendezvous-based	Multiple mobile sinks	False	Centralized	Network cost optimizing

**Table 2:** Parameters for PSO and ACO.

Parameter Name	Parameter Value
Number of SPs (spn)	15
Number of particles in PSO (pn)	50
Inertia coefficient of particles in PSO (λ)	0.7
Weight coefficients of local update in PSO (α)	0.4
Weight coefficients of global update in PSO (β)	0.6
Number of ants in ACO (m)	30
Control factor for pheromone concentration in ACO (α)	2
Control factor for inspired factor in ACO (β)	3
Volatilization rate of pheromone in ACO (η)	0.5

**Table 3:** Network parameters.

Parameter Name	Parameter Value
Length of the sensor field (L)	400 × 400 m
Number of sensors (N)	200
Communication range of sensors (r)	60 m
Primary energy of each sensor ($E_{0}$)	0.05 J
Data generation rate of each sensor ($v_{s}$)	1 bit/s
Capacity of each sensor ($C_{s}$)	2 MB
Moving velocity of the mobile collector ($v_{m}$)	2 m/s
Number of SPs (nsp)	[12,13,14,15,16]
Sojourn time for each SP ($E_{0}$)	5 s
Energy consumption of transmission circuit ($E_{elec}$)	50 nJ/bit
Amplifier parameter for free-space model ($\varepsilon_{fs}$)	10 pJ/bit/m^2^
Amplifier parameter for multi-path model ($\varepsilon_{mp}$)	0.0013 pJ/bit/m^4^

## Data Availability

The data that support the findings of this study are available from the corresponding author upon reasonable request.
